# The Sensory and Emotional Response to Different Tableware Materials

**DOI:** 10.3390/foods14183151

**Published:** 2025-09-09

**Authors:** Ana Pantović, Ilija Djekić, Tanja Petrović, Nikola Tomić

**Affiliations:** Faculty of Agriculture, University of Belgrade, Nemanjina 6, 11080 Belgrade, Serbia; idjekic@agrif.bg.ac.rs (I.D.); tpetrovic@agrif.bg.ac.rs (T.P.); tsnikola@agrif.bg.ac.rs (N.T.)

**Keywords:** tableware, cutlery, emotions, acceptance, perception

## Abstract

The high environmental impact caused by the accumulation of single-use plastic calls for measures to curb this problem, from a ban on single-use plastic tableware to the production of a wide range of biodegradable and reusable products. The aim of this study was to investigate how tableware made of different materials affects consumers’ sensory perception and emotional and hedonic responses when eating the same meal. In this study, four types of meals of animal or plant origin were selected for the experiments, which were served warm or cold. Accordingly, four groups of university students were instructed to taste the corresponding meal while using three sets of tableware made of different materials: polypropylene, wood/cardboard, and a stainless steel/ceramic/glass control set (regular set). Overall, the results suggest that the use of regular tableware elicited a positive emotional profile, while the use of disposable, wooden, and plastic tableware elicited negative emotional responses, which is consistent with the acceptability of the meal samples—regular tableware received higher ratings, while both types of disposable tableware received lower ratings. Finally, the material of the tableware only led to changes in odor and flavor perception when warm-served meals were sampled—higher intensities were reported when students used the regular tableware sets. Wooden cutlery imparted an atypical woody flavor to the meals, regardless of the type of meal.

## 1. Introduction

The trend of “externalizing food preparation” (i.e., reliance on food preparation and consumption outside the home) has been increasing in Europe and the United States [1,2], driving growth in both ready-to-eat meals and the catering industry. The primary drivers of this shift are the growing availability and convenience of ready-made meals. Although this type of meal offers convenience, environmental pollution of discarded packaging, particularly from plastic material, still remains a concern [3]. Everyday disposable items, such as paper coffee and tea cups, cutlery (commonly used at large and small gatherings), contribute significantly to this issue, accumulating in oceans and landfills [4]. Plastic waste does not biodegrade [5] and only about 9% is recycled [6], while a small portion is incinerated, thus leaving huge amounts of waste ending up in landfills or the environment, potentially posing negative health and environmental consequences [7,8,9]. Therefore, it is essential to tackle these problems with adequate measures focused on decreasing plastic consumption and promoting more sustainable alternatives. The European Union (EU) aims to decrease packaging waste by 15% by 2040 through various waste management strategies and by enabling the creation and development of fully recyclable packaging by 2030 [10]. Additionally, single-use plastic plates, cutlery, and straws will be prohibited [11]. In parallel, it is worth mentioning that the new Trump administration in the US is opposing this trend overseen in several countries worldwide [12]. Along with this ongoing debate over banning single-use tableware, a wide range of biodegradable and reusable alternatives have been introduced to the market [13]. However, there is still a lack of sensory research on the performance of these alternatives, which could be valuable in facilitating their broader adoption.

Human sensory perception primarily relies on five senses: vision, olfaction (smell), gustation (taste), touch, and hearing [14]. Since different senses shape human sensory perception, attempts have been made to investigate the presence of cross-modal interactions between them. Additionally, when we taste a food or beverage, we first form an expectation about its flavor and how much we anticipate enjoying it [15]. This psychological concept in this context is known as “sensation transference.” Researchers suggest that our perceptions of one stimulus can, at least partially, influence our perceptions of another [16]. For instance, the sense of smell is influenced by other sensory inputs. In the case of vision and olfaction, matching visual cues can enhance odor recognition [17] and alter perceived odor intensity [18]. Further, if we perceive the cup we are drinking from as high-quality or aesthetically pleasing, these impressions may transfer to our evaluation of the coffee itself [19].

This has paved the way for research in consumer science regarding the link between tableware and food perception when varying tableware attributes. For example, there has been a growing interest in exploring how the drinking vessel—whether a cup or mug—affects the coffee-drinking experience [20]. Recent research has examined the impact of cup shape [21], color [21,22], and texture [21] on taste and tactile judgements. Findings from multiple studies now confirm that the vessel in which coffee is served can significantly influence the drinking experience for both experts and consumers. This was confirmed in the case of wine as well, as it was observed that the shape of a wine glass can influence flavor release, and thus affect the perceived bouquet of the wine [23].

Another important factor that influences sensory perception of foods is the material of tableware. For example, a soft drink was rated as the most acceptable when consumed from a glass cup and the least acceptable when tasted from a styrofoam cup. Additionally, styrofoam, paper, and polystyrene negatively affected the overall acceptability of the soft drink [24]. Perception of cream samples with sweet, sour, bitter, salty, and plain taste was also found to be influenced by the difference in metal material the spoons were made of. Zinc and copper spoons impaired metallic and bitter taste, and enhanced the dominant taste of each cream to varying degrees. Surprisingly, the metallic flavor from the copper and zinc spoons did not significantly affect the overall pleasantness of the samples [25]. Although inert and tasteless, some metals may transfer flavors to food, thus enhancing/inhibiting the food’s natural tastes, consequently either improving or diminishing the overall sensory experience.

Along with the sensations that happen in the mouth and shape the sensory perception of food, people’s judgments of food perception can also be influenced by other sensory cues, such as haptic input, resulting from direct contact with the food or indirect contact with the product packaging of tableware [26]. For instance, the texture of the container that the participants held in their non-dominant hand influenced their ratings of certain texture attributes of yoghurt and biscuit samples, suggesting that non-diagnostic haptic cues (those that objectively should not affect the perception) have a role in shaping food perception [26]. Further, meals served with natural wooden tableware were rated more favourably than those served on plastic tableware (both before and after consumption) [27]. Changes in the haptic qualities of a product’s packaging, or the utensils used to consume it, could significantly influence a consumer’s appraisal of the product’s quality and their overall experience [23,28,29].

Food can also impact individuals’ emotional states. Studies have shown that taste quality can influence affective responses [30]. For example, research has demonstrated that newborns experience positive affect when exposed to sweet solutions and negative affect to bitter solutions [31]. While most studies focus on general valence (pleasant versus unpleasant experiences), the few studies that have measured distinct emotions show that tastants and food stimuli can also affect the specific nature of one’s emotional state. For instance, the study by Pramudya et al. (2021) demonstrated that various drinking straw materials influence emotions and sensory perceptions of cold tea [32].

In light of the published research, this study aims to investigate how tableware made of different materials influences sensory perception and evokes emotions in consumers while consuming the same meal.

## 2. Materials and Methods

### 2.1. Food and Tableware Samples

Four types of meals of animal or plant origin were selected for the trials, which were to be served warm or cold. The animal-based meal served warm was a sterilized, ready-to-eat pork stew, while the warm plant-based meal was a sterilized, ready-to-eat green pea meal, both available in 400 g cans and made by a local producer. The cold-served animal-based meal was slices of fermented sausage and semi-hard cheese, while the cold-served plant-based meal consisted of tomato and cucumber slices, all purchased from a local supermarket. Warm-served dishes were reheated in a pot containing water, and the dishes had been preheated to 50–55 °C for 10 min. In addition to the meals, participants were also provided with water served in cups made of three different materials.

Three sets of tableware made from different materials were used in the consumer tests. The less environmentally friendly tableware set consisted of the following items made of polypropylene (PP): spoon, fork, knife, plate, and cup. The environmentally friendly tableware set consisted of a fork, a knife, a spoon made of birch wood, and plate and cup made of paper (cardboard). Deep cardboard plate is coated with 12 micron polypropylene to enable the water resistance of the served meal, while the shallow plate is made of pure Cromo cardboard (Table 1). The control set consisted of stainless steel fork, knife, and spoon; ceramic plate; and glass cup. The general characteristics of the tableware used are given in Table 1.

### 2.2. Consumer Testing 

The consumer tests involved 247 volunteers, consisting of students aged between 18 and 30. Generation ‘Z’ (Gen Z), is a generation that was born between 1995 and 2010 and currently represents one third of the world’s population [33]. Due to their contribution to global consumption patterns, they have the potential to impact food consumption patterns [34]. In parallel, this generation has an emphasized sustainability awareness, which may affect future development of new, sustainable tableware materials [35]. To avoid any bias in terms of tableware materials and avoid preference towards certain tableware materials, they were selected randomly on the basis of their general attitude towards the test meals and no possible aversion to the meals without mentioning tableware materials. The plan was to use different groups of approximately 60 subjects for different test meals rather than using a larger group to evaluate all meals, so as not to transfer the same tendencies, preferences, and attitudes from one meal to another. Within each group, students evaluated the same meal with the three sets of tableware in separate sessions. In this way, all possible combinations of the four types of meals and the three types of tableware were included in the experimental series (a full factorial design). Within each student group, samples of the corresponding meal served with different types of tableware were presented in random order. When tasting the meals served cold (plant-based and animal-based), consumers were instructed to cut the dish with a knife and fork. It was important to ensure that consumers used the appropriate type of cutlery depending on the meal offered (i.e., warm-served meals were eaten with a spoon, while cold-served meals were eaten with a fork and knife). The tests were conducted in the following order. First, participants provided personal information (age, gender) and rated common disposable tableware materials according to their suitability for group celebrations and large events. Participants were then instructed to assess each meal with one of the tableware sets on offer in random order and to complete the questionnaire on their emotional reactions. Finally, they rated the acceptability and intensity of selected sensory attributes of the meals for each of the three tableware sets offered. Participants who did not provide demographic information were not included in the analysis. This resulted in a total of 242 participants, who were divided into four groups, according to the type of meal tested: Warm animal-based meal—Pork stew (*n* = 59), Warm plant-based meal—Peas (*n* = 61), Cold animal-based meal—Cheese and meat (*n* = 62), Cold plant-based meal—Salads (*n* = 60).

#### 2.2.1. Ranking of the Tableware Materials

On the first sheet, participants were asked to fill in their personal details (gender and age group) and rank the materials they found most and least acceptable for disposable tableware at large events. Materials of interest included paper, soft plastic, hard plastic, and wood. 

#### 2.2.2. Assessment of Emotional Responses

The list of moods and emotions comprised 28 selected terms (Supplementary Table S1) from the Consumer Classification of Emotions table by King and Meiselman (2010) [36]. A 4-point category scale was used to assess the intensity of the evoked emotions, corresponding to the following values: 0 = “not at all”; 1 = “slightly”; 2 = “moderately”; and 3 = “very intensely”. The emotional response data obtained were later converted into a binary form. This procedure was used instead of classical CATA (Check-All-That-Apply) method, because by rating the intensity of each emotion listed, whether it was evoked or not, participants were forced to go through the entire list of terms without skipping some of them, which can easily happen when using the check-only procedure [24].

#### 2.2.3. Acceptance and Attribute Intensity Testing

The meals were assessed for liking using a 9-point hedonic scale (1 = ‘extremely dislike’, 5 = ‘neither like nor dislike’, 9 = ‘extremely like’) for the following attributes: ‘taste of the meal’, ‘dishes’, ‘cutlery’, and ‘served meal’. The attribute ‘served meal’ stood for the overall impression of the meal, which included the entire arrangement together with the tableware (plates, cups, and cutlery).

The intensity of the five selected sensory attributes (overall odor, saltiness, flavor of spices, atypical flavor, overall flavor) was assessed using a 7-point intensity scale.

### 2.3. Descriptive Sensory Testing of Tableware Sets

Eight staff members from the University of Belgrade, experienced in descriptive analysis, conducted a descriptive sensory evaluation of tableware samples made of different materials. The tableware items evaluated were: spoon, fork, and knife (made of wood, plastic, and steel), cup (made of paper, plastic, and glass), and plate (made of paper, plastic, and ceramic). Following the procedure described by Heymann, King, and Hopfer (2014) [37], a consensus list was created after several consecutive sessions conducted with the tableware items. The list consisted of nine attributes for spoon, fork, and knife, and eight attributes for cup and plate (Table 2). The assessment was conducted in one repetition, using a similar method to the flash profile method [38]. Panellists used an unstructured 15 cm line scale to rate intensity in their own way. They rated the attributes by comparing the tableware items made of different materials directly with each other. 

### 2.4. Statistical Analysis

The data on the intensity of the emotional reaction were first converted into binary values as follows: scores of 1, 2, and 3 were assigned the value ‘1’ (indicating a ‘selected/evoked’ emotion), while scores of 0 were assigned the value ‘0’ (indicating a ‘not selected/not evoked’ emotion). In addition, another two variables were defined (‘like it’ and ‘dislike it’) by converting the hedonic ratings of the “served meal” into binary data. For the variable ‘like it’, the values 6, 7, 8, and 9 were coded as ‘1’ (meaning ‘selected’), while the values 1, 2, 3, 4, and 5 were coded as ‘0’ (meaning ‘not selected’). For the variable ‘dislike it’, the values 1, 2, 3, and 4 were coded as ‘1’ (‘selected’), while the values 5, 6, 7, 8, and 9 were coded as ‘0’ (‘not selected’).

The variables describing the emotional responses that were included in the further statistical analysis were selected based on a 10% cut-off for the total number of occurrences for each meal, and this selection was made separately in each of the four groups. This means that the number of emotional response variables differed between the groups, since the following tests were conducted separately in each of the groups. The selected emotional response variables were analyzed using Cochran’s Q test, followed by multiple pairwise comparisons using Sheskin’s critical differences procedure to identify the emotions that differed significantly between the sets of tableware (with a significance threshold of α = 0.05). The emotional response variables that reached statistical significance after applying Cochran’s Q test were then subjected to correspondence analysis (CA).

The raw hedonic data and attribute-intensity data were subjected to one-way Analysis of Variance (ANOVA) (with assessors as a random factor) and Tukey’s HSD test for multiple pairwise comparison (α = 0.05), for each of the four defined groups/meals separately. Additionally, the dependent samples two-tailed t-test was applied to compare ‘taste of the meal’ and ‘served meal’ hedonic data for each type of meal used (α = 0.05).

The rank data were subjected to the Friedman-type statistic for rank data, followed by the LSD multiple comparison procedure for rank sums from a complete block design (α = 0.01) [14].

The descriptive sensory data were first standardized across assessors [39] and then ANOVA was performed (with ‘assessors’ as a random factor), followed by Tukey’s HSD multiple pairwise comparison test (α = 0.05) to identify the attributes that significantly differentiate tableware items made of different materials. The raw descriptive data of the significant sensory attributes were analyzed using Generalized Procrustes Analysis (GPA) to obtain a rescaled consensus data matrix, which was then subjected to Principal Component Analysis (PCA) of the correlation matrix.

Statistical software used: R studio version 2024.04.2 [40], SPSS Statistics 17.0 (SPSS Inc., Chicago, IL, USA), and XLSTAT 2023.2.1414 (Addinsoft, Paris, France).

## 3. Results

### 3.1. Survey of Tableware Materials

All students who took part in the sensory trials were asked to give their opinion on the suitability of disposable tableware made of different materials at large events. A total of 242 participants (163 women and 79 men between the ages of 18 and 30) ranked four types of materials: hard plastic, soft plastic, wood, and paper. Hard plastic appeared to be statistically the most acceptable and paper the least acceptable (*p* < 0.01) material for disposable tableware used at large parties and events. No statistically significant difference was found between soft plastic and wood (Supplementary Table S2).

### 3.2. Emotional Responses

The main purpose of including emotional responses in the experiments was to investigate whether there were any regularities in the distribution of positive and negative emotions in relation to the use of different types of eating utensils, without going into psychological explanations for emotional reactions. Therefore, the focus was placed on the consumers’ reactions to the entire meal served. Participants were asked to rate their emotional response on a scale of 0 to 3 for 14 positive and 14 negative emotions when they tasted the meal offered with three different sets of tableware. After converting these responses into binary data, the emotional responses selected at less than 10% of the total number of occurrences for each of the four meals tested were excluded from further statistical analysis. The remaining variables were then tested for significance in distinguishing between the samples. The results are presented in Table 3.

**Figure 1:**
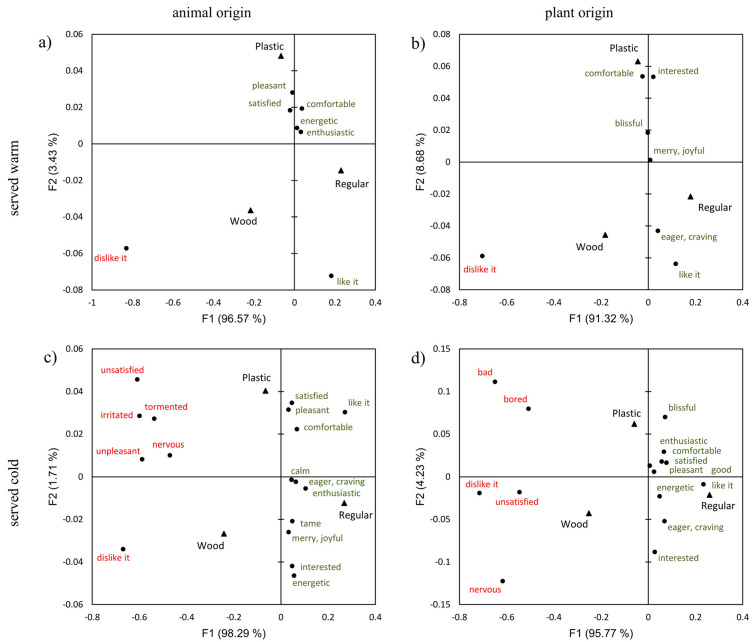
Emotional and hedonic responses associated with the consumption of four different meals with three different tableware sets. The biplot shows the results of the correspondence analysis applied to the data of 242 participants divided into four different groups, (**a**) warm animal-based meal—Pork stew (*n* = 59), (**b**) warm plant-based meal—Peas (*n* = 61), (**c**) cold animal-based meal—Cheese and meat (*n* = 62), (**d**) cold plant-based meal—Salads (*n* = 60). Red corresponds to negative, and grey to positive emotional responses.

There was a total of 10 positive and 9 negative emotions (and the two hedonic variables converted to binary data) across all four groups, which were found to significantly discriminate among the samples (α = 0.05) and were further included in the CA. The results of the CA conducted on the selected emotional and hedonic response frequencies are presented in Figure 1.

The arrangement of both positive and negative emotions in relation to the positions of the tableware sets in the biplots is very similar for all types of meals served (Figure 1). In each case, the first factor extracted explained more than 90% of the variance in the original contingency datasets, suggesting that the main differentiation between the samples can be observed across the horizontal axes. The statistical analysis presented in Table 3 also confirms this assertion. The wooden tableware sets were positioned on the left sides of the biplots, mainly towards negative emotions, while the regular sets appeared on the right sides, mainly towards positive emotions. The plastic sets were positioned in the biplots mainly near the origin, in the middle between the former two samples, indicating similar emotional responses as for wooden and/or regular tableware, as clearly shown in the results of the statistical significance analysis in Table 3. For the wooden tableware, but also for the plastic sets, the results show that a greater number of negative emotions were elicited when eating the cold-served meals, both animal- and plant-based, than the warm-served samples. Tasting the cold-served ‘meat and cheese’ and salads with disposable utensils, primarily the wooden sets, was associated with negative emotions such as ‘unsatisfied’, ‘nervous’, ‘unpleasant’, ‘bad’, ‘bored’, ‘irritated’, and even ‘tormented’, which presumably influenced participants' liking behavior. The proportion of participants who disliked the meals of all types served with wooden or plastic tableware ranged from approximately 20% to 42% (26.2–41.9% for wood and 18.0–27.4% for plastic), while these proportions were less than 5% for regular tableware in each case (Table 3). When looking at the different types of tableware used, negative emotions were not found to be statistically significant (*p* > 0.05) in the case of warm-served pork stew and peas tasted with spoons, indicating that these types of meal and tableware combinations did not negatively affect emotional state, regardless of preference behavior. In contrast to wood and plastic, the use of regular tableware for tasting the meals elicited mainly positive emotions such as ‘pleasant’, ‘satisfied’, ‘comfortable’, ‘joyful’, ‘enthusiastic’, ‘interested’ and ‘eager’, which could probably be a reason why the proportion of those who liked the ‘served meals’ ranged from 88.7% to 95.1%.

### 3.3. Results of Acceptance and Attribute Intensity Testing

The acceptance tests were conducted in the following order, gradually shifting attention from the food to the tableware: first, participants were asked to taste the food and rate the acceptability of the ‘taste’; then they were asked to assess the acceptability of the tableware; and finally, to rate the entire meal served, taking into account the accompanying tableware. To investigate whether tableware made of different materials can influence the acceptability of the tasted meal, the hedonic ratings of the taste were compared with the ratings of the entire meal served. The assumption was that the influence of tableware on acceptability would be insignificant if the focus was solely on ‘taste’.

The acceptability scores for the dishes and cutlery showed a large difference between the regular (ceramic, glass, metal) and the disposable (wood and plastic) tableware, which could reflect eating habits. The type of meal had no effect on the acceptability of the dishes used, while the ratings for the cutlery were statistically significantly lower (*p* < 0.05) for cold-served meals than for warm-served meals, as shown by the two-way ANOVA (results not presented). The mean hedonic scores for the disposable dishes and cutlery did not differ significantly (*p* > 0.05) by the individual attributes rated and fell into either the “dislike” or “neither like nor dislike” category with scores ranging from 3.1 ± 2.5 and 5.0 ± 2.3, while the ‘regular’ tableware was rated almost the highest on the 9-point scale at 7.9 ± 1.5 to 8.2 ± 1.8 (Table 4).

According to the mean hedonic scores for ‘taste of the meal’, which ranged from 6.2 ± 2.2 to 7.6 ± 1.8, the participating student groups liked the taste of all meals served. There were no statistically significant differences in the hedonic ratings for ‘taste’ between the use of regular and plastic tableware, but the scores for the use of wooden tableware were statistically significantly lower than the corresponding scores for regular tableware in every case except peas (*p* < 0.05). This decrease is most likely due to the contribution of wooden tableware to the flavor of the meals, as it introduces an atypical flavor. In the intensity tests of selected sensory attributes, it was observed that the wooden tableware imparted an atypical flavor to the meals consumed (Table 5). When looking at the acceptability results for ‘taste of the meal’ and ‘entire meal served’, the statistical analysis showed that all hedonic ratings for the use of wooden and plastic tableware for the entire meal served were significantly lower (*p* < 0.05) than the corresponding ratings for the acceptability of the ‘taste of the meal’. However, it should be noted that all mean hedonic scores for the entire meal served for the wooden and plastic tableware fell into the “neither like nor dislike” category. In contrast, when using regular tableware, there was a statistically significant increase in hedonic scores for the entire meal compared to the corresponding scores for the ‘taste of the meal’ for all meal samples, except for the cheese and sausage slices, whose ratings did not differ significantly. These results, together with the acceptability ratings for the dishes and cutlery used, indicate that the tableware used in the trials influenced the acceptability of the meals served: the disposable tableware negatively, the regular tableware positively.

The potential influence of tableware made of different materials on the perception of the food consumed was tested by evaluating five selected sensory characteristics using a 7-point intensity scale: overall odor, saltiness, flavor of spices, atypical flavor, and overall flavor. The salad, being the cold-served meal in the study, was excluded from this experiment as it was served fresh, without salt, spices, dressings, or other added ingredients. The results are shown in Table 5.

In general, it appears that the perception of the odor and flavor attributes evaluated, except for ‘atypical flavor’, was not influenced by the tableware made of different materials for the cold-served sausage and cheese slices eaten with a knife and fork. The differences were only found regarding the warm-served meals. The attribute ‘flavor of spices’ was also not influenced, regardless of the type of meal served. Wherever there was a statistically significant (*p* < 0.05) difference in the perception of saltiness and overall odor and flavor, the meals (both the warm-served pork stew and the peas) were rated with higher intensity scores when regular tableware (ceramic plates and stainless-steel cutlery) was used, compared to the wooden and plastic sets. No statistically significant differences were found in the perception of these attributes between the use of wooden and plastic tableware.

An ‘atypical flavor’ was noted in all meals served when the wooden cutlery was used for tasting. According to the comments of some test participants, this atypical flavor was perceived as that of wooden cutlery in general. Since the wooden forks and spoons came into direct contact with the tongue and/or some surfaces in the oral cavity, it is very likely that this atypical flavor came directly from the cutlery and not from the “tainted” food as an intermediate to which the atypical flavor was transferred.

### 3.4. Descriptive Analysis

**Figure 2:**
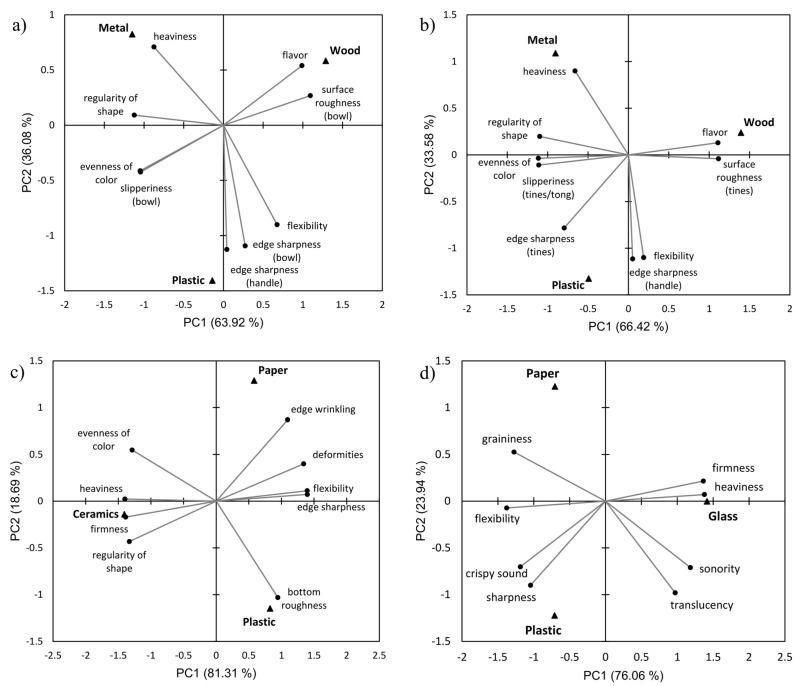
Sensory maps of the tableware made of different materials used in the study, derived from the descriptive sensory analysis: (**a**) spoons, (**b**) forks, (**c**) plates, and (**d**) cups. The biplots show the results of the Generalized Procrustes Analysis and Principal Component Analysis applied to the raw descriptive sensory data.

The descriptive sensory evaluation of the tableware used in the study was conducted in five separate sessions for spoons, forks, knives (results not shown), plates, and cups. The results of the ANOVA and subsequent multiple pairwise comparisons applied to the standardized descriptive data are shown in Supplementary Tables S3–S6. The attributes that did not show statistically significant differences between tableware items made of different materials were excluded from dimension reduction analysis (GPA followed by PCA). These results are shown in Figure 2.

Very similar, almost identical sensory maps were obtained for spoons and forks (Figure 2a,b), which was to be expected as the items came from the same cutlery sets. Stainless steel cutlery was characterized by pronounced heaviness, regularity of shape, and evenness of color, as well as low edge sharpness of the handle and low flexibility (*p* < 0.05). The edge sharpness of the handle and bowl/tines and flexibility were most pronounced in the plastic cutlery, while the surface roughness and slipperiness were at the same level as in the stainless-steel items. Wooden cutlery was characterized by a pronounced surface roughness and low levels of bowl/tines edge sharpness, slipperiness, heaviness, regularity of shape, and evenness of color. Probably the most important point, however, is that wooden cutlery has a specific woody flavor, which was also registered in the attribute intensity tests with consumers/students (Table 5).

For the plates and cups, sensory profiling revealed similar biplots, i.e., sensory maps (Figure 2c,d). Ceramic plates and glass cups were characterized by pronounced heaviness and firmness, as well as a lack of edge sharpness and flexibility. The presence of deformities, edge sharpness, and surface roughness was more pronounced in the plastic and cardboard dishes than in ceramic and glass. Compared to the ceramic plates, the plastic and cardboard were light and flexible and showed less regularity of shape and evenness of surface color. The plastic cup was also assessed as light, flexible, and with the most intense crispy/rustling sound when squeezed in the hand. Cardboard and plastic cups also had a more pronounced grainy surface below the rim, compared to the smooth surface of the glass.

## 4. Discussion

It is now known that a change in tableware with different tactile and visual properties (weight, shape, size, mechanical texture, transparency, color, etc.) can affect the perceived flavor of the food served and the emotional and hedonic reactions to the meals consumed, thus altering consumption behavior [28,41,42]. In the present study, the influence of sensory cues emitted by tableware on the perception, emotional profile and acceptability of food through the stimulation of the anticipatory and consummatory senses (before and after the food enters the mouth, respectively) was investigated by using three sets of tableware in a full factorial design with four types of meals, each evaluated by a separate group of university students. Apart from the effect that wooden cutlery generally had on the perception of the atypical (woody) flavor, the influence of the tableware used on flavor perception was only statistically significant for the warm-served meals (pork stew and peas), compared to the cold-served deli meat and cheese (Table 5). The pork stew and peas were the meals with a liquid phase and had to be eaten with spoons, while the cold-served deli products were tasted with forks and knives. The fuller and longer contact of the spoon with the lips and tongue during eating as opposed to the use of forks could be a reason for these different effects of tableware on the flavor perception of the meals. Taking this assumption into account, it could be said that visual cues had no influence on flavor perception in this case. The ‘salty taste’, the ‘overall flavor’, and partially also the ‘flavor of spices’ of the warm-served meals were perceived with higher intensity when metal spoons were used, in contrast to wooden and plastic cutlery. Compared to wooden and plastic spoons, the metal spoon was perceptibly heavier, with a higher regularity of shape (Supplementary Table S3). This pattern of a heavier spoon influencing a higher perceived intensity of a flavor note has also been reported in some previous studies. In the experiment with yoghurt tasted with plastic teaspoons or tablespoons whose weight was controlled by adding weights in the handles, Harrar and Spence (2013) [42] found that the sweetness of the yoghurt varied with both the spoon size and the spoon weight. The yoghurt tasted with the heavy spoon was rated as the sweetest. They linked the influence of spoon size on perceived sweetness to taste expectations, since small spoons are typically used for stirring honey or sugar into tea or coffee or for desserts, as opposed to larger tablespoons, which are more commonly used for soups and cooked meals. Although the correlation between the weight and sensory perception intensity was not directly assessed in this study, the observed patterns are consistent with prior cross-modal perception literature, suggesting that such association may explain the observed findings.

The influence of the woody flavor introduced by wooden cutlery on the perception of the other flavor notes evaluated is unclear and cannot be explained by the results obtained (Table 5). Although it could be assumed that the woody note influenced the suppression of ‘saltiness’ and ‘flavor of spices’ in some way, as a significant decrease in the perceived intensity of the two notes was observed in contrast to the use of regular/metal cutlery (*p* < 0.05), the order of perceived intensities of ‘overall flavor’ (higher with the metal spoon than with the wooden and plastic spoons) and the fact that there were no significant differences in ‘saltiness’, ‘flavor of spices’ and ‘overall flavor’ between wooden and plastic tableware do not support this assumption. It is more likely that the weight of the cutlery ultimately influenced the perceived flavor of the meals.

Regular, reusable tableware consistently elicited positive emotional responses to the meals, while disposable wooden cutlery was predominantly associated with negative emotions, especially when used for the cold-served meals. The same pattern was seen in the acceptance of the meals. All types of meals tested were significantly better accepted when using regular tableware than when using wooden and plastic sets. This was not just a matter of statistical significance, but the mean hedonic ratings for the ‘entire meals’ served with the regular tableware (7.5 ± 1.2–7.8 ± 1.7; Table 4) were in the ‘like’ range, with the proportion of those who liked the meals ranged from 88.7% to 95.1% (Table 3), while the mean ratings for the meals served with wooden/paper and plastic tableware fell into the “neither like, nor dislike” category (up to 42% of those who disliked the meals; Table 3). The differences between the hedonic ratings for ‘taste of meal’ and ‘entire meal served’ observed for each type of meal tested (Table 4) suggest that regular (metal/ceramic/glass) tableware positively influenced meal acceptability, while wooden and plastic tableware had a negative influence. The main reasons for these results could lie in eating habits and expectations about what type of tableware should be used for what type of meals, as well as the interaction with the tableware and its sensory properties, which could also play an important role. The use of disposable tableware in everyday life would not be financially viable for households, so it can be assumed that most people are used to reusable tableware, which is usually made of metal, ceramic, or glass. The regular tableware used in this study was primarily characterized by a distinct heaviness and firmness, as well as a lack of flexibility and edge sharpness (Figure 2), all of which can be considered characteristics that positively influence suitability for serving food and ultimately shape consumers’ emotional and hedonic reactions. The ‘firmness’ and ‘material from which the cups were made’ were found to be very important characteristics of disposable cups for younger adults, such as university students [24]. Unbranded cola from a heavier can was rated as tasting better than from a lighter can [43], while heavier bowls increased the liking of yogurt [44]. On the other hand, the wooden cutlery released a typical woody flavor on contact with the surfaces of the oral cavity and had a pronounced surface roughness with a low degree of slipperiness, heaviness, and regularity in shape, whereas paper plates and cups were completely flexible and not firm (Figure 2). It may be that these characteristics negatively affect the eating experience and evoke negative emotional and hedonic responses.

Furthermore, the results of the survey distributed among the university students indicate that they prioritize the strength, durability, and functionality of tableware materials, which underscores the importance of material properties when selecting disposable tableware. Hard plastic was ranked as the most suitable material for disposable tableware for large events, followed by wood and soft plastic, and with paper at the bottom as the least suitable (Supplementary Table S2). Since these four materials were the only options presented in the survey, participants’ responses might have been influenced by their prior experiences with the sensory characteristics of these materials, and by their expectations that disposable tableware should have characteristics similar to those made of hard materials such as metal, ceramic, or glass. Pramudya et al. [32] also reported the same trend of differences in emotion profiles with those in acceptability when consuming cold tea with drinking straws made of different materials. The use of copper straws elicited positive emotions such as ‘satisfied’, ‘free’, ‘adventurous’, and ‘enthusiastic’, which also led to higher acceptability, in contrast to paper straws, which were least liked and were associated with negative emotions such as ‘disgusted’. The importance of hedonic impressions in the expression of emotions triggered by food and drink has also been reported in previous studies [36,45], although the intensity of emotions and acceptability do not always follow the same trend [36].

In a study conducted in Japan, which examined the influence of plastic or natural wooden tableware on the perception of and satisfaction with ready-to-eat meals, the authors found that both sets of tableware had no influence on the perception of the taste and texture of the meals, but that natural wooden tableware increased satisfaction with the meals [27]. Meals served on natural wooden tableware were rated more positively in the pre- and post-tasting evaluations than those served on plastic tableware. In the present study, plastic cutlery showed an ambiguous emotional profile that shared both positive and negative attributes with regular and wooden tableware, respectively (Figure 1). No significant differences were found between the influence of plastic and wooden/paper tableware, both in the perception of selected sensory characteristics (Table 5) and in the hedonic responses (Table 4) to the tested meals served warm or cold. There are several reasons for this possible discrepancy between the two findings. First, in the former study, the authors used hardwood plates in addition to wooden cutlery, which cannot be considered as disposable (single-use) tableware, whereas in the present study, flexible paper plates (and cups) were used. These two tableware sets (used in the Japanese study and in our study) inherently differ in the properties of materials used for their production. However, the former study did not assess the descriptive attributes of the tableware made of different materials that they used in their study. Further, the paper plates we used in our study were very similar in certain textural properties to the plastic plates. Both were primarily flexible and not firm (Figure 2c), which could be a reason for their negative influence on the hedonic response to the tested meals (Table 4), but also on the emotional response (Figure 1). The survey conducted in one of our previous studies [24] on the importance of characteristics of disposable cups revealed that properties such as ‘firmness’ and ‘material from which the cups were made’ were very important for younger adult consumers, such as university students, in addition to ‘smoothness of the rim’ and the ability to ‘not transfer flavor to the beverage’ which were ranked as most important. In addition to the influence of eating habits, the present study suggests that the achieved negative emotional and hedonic response to the food may be influenced by the sensory characteristics of the disposable plastic and wood/paper tableware. Secondly, in the Japanese study, the authors only compared plastic and natural wooden tableware, while the present study also included ‘regular’ sets with stainless steel cutlery, ceramic plates, and glass cups. The reason for the similar emotional and hedonic responses, as well as the perception of the intensity of selected sensory characteristics in the case of plastic and wood/paper tableware compared to the regular sets, where the responses differed significantly from the previous two, could therefore be attributed to the psychological effect known in perceptual psychology as convergence or condensation. The explanation for this effect is as follows: “A group of items may seem more similar to each other when they are in the presence of an item that is very different from that group” [46,47]. Finally, the variability in the results might result from the cultural differences that exist between the two countries.

### 4.1. Limitations of the Study

This study has several limitations. Firstly, the participant group was relatively homogeneous (“Z” generation) and did not include some other populations (such as families, elderly people), which may limit the generalizability of the results. Secondly, the study included only a limited number of dishes, potentially restricting the applicability of the findings to other meal types or culinary contexts worldwide. Third, there was a lack of control over the temperature of the ‘real hot’ meals served, but included warm-served meals, which could have influenced participants’ perceptions and responses. The selection of biodegradable materials in terms of selecting common materials opposed to newly developed materials such as polylactic acid or starch-based composites could lack generalizability, as the new materials may have unique behaviors or characteristics that could differ from common materials.

### 4.2. Further Research

Future research could explore the connection between dietary habits of elderly population and preferences towards new types of tableware. By extending research to encompass broader consumer demographics, dietary habits, and advanced tableware material science, it will enable better understanding of current trends and needs. Additionally, expanding the research to “real-world” rather than just laboratory settings would yield insights more representative of everyday consumption contexts. These studies can contribute to more inclusive, sustainable, and enjoyable eating experiences reflecting the diversity and creativity of modern society.

## 5. Conclusions

Overall, this study found that emotional responses were consistently positive when students used regular, reusable (metal/ceramic/glass) tableware when tasting the meals, while the opposite was observed when they used disposable wooden/paper and plastic cutlery. This was particularly true for meals served cold. Furthermore, the hedonic ratings showed that both types of disposable tableware had a negative impact on the acceptability of the meal served, while the regular tableware had a positive impact. Finally, the differences in the intensity of odor and flavor attributes associated with the tableware materials were only observed for meals served warm. Meals consumed with regular tableware were perceived as more intense in terms of ‘overall odor’, ‘salty taste’, and ‘overall flavor’ than meals consumed with disposable tableware. It could be that the interaction with the tableware and its sensory characteristics, but also eating habits and expectations about what type of tableware should be used for what type of meals, were the main reasons for such an influence of the tableware used in this study on the meals served. The reasons for similar sensory perceptions, and emotional and hedonic responses to the meals served in the case of the plastic and wooden/paper tableware compared to the metal/ceramic/glass tableware sets, could be attributed to the psychological effect known as convergence or condensation, where the presence of an object that is very different from a group of other objects makes that group appear more similar to each other, which would not be the case in its absence.

## Figures and Tables

**Table 1:** Characteristics of the tableware items used for the experiment.

Tableware Item	Material	Weight (g)	Height/Outer Diameter (cm)
The regular set of tableware			
Spoon	Steel	35.76 ± 0.40	17.14 ± 0.23
Fork	Steel	37.14 ± 0.21	20.1 ± 0.10
Knife	Steel	81.42 ± 1.32	23.48 ± 0.11
Plate (deep)	Ceramic	411.27 ± 10.35	19.98 ± 0.04
Plate (shallow)	Ceramic	557 ± 8.67	25.08 ± 0.08
Cup	Glass	259.72 ± 1.57	7.56 ± 0.05
The wooden set of tableware			
Spoon	Wood	2.65 ± 0.42	15.9 ± 0.14
Fork	Wood	2.49 ± 0.19	15.8 ± 0.12
Knife	Wood	2.65 ± 0.23	16.46 ± 0.11
Plate (deep)	Paper	12.70 ± 0.01	19.68 ± 0.19
Plate (shallow)	Paper	11.01 ± 0.06	21.68 ± 0.13
Cup	Paper	4.40 ± 0.03	6.9 ± 0.14
Plastic set of tableware			
Spoon	Plastic	2.44 ± 0.16	16.3 ± 0.32
Fork	Plastic	2.22 ± 0.14	16.3 ± 0.19
Knife	Plastic	2.24 ± 0.03	16.42 ± 0.16
Plate (deep)	Plastic	8.69 ± 0.34	20.38 ± 0.16
Plate (shallow)	Plastic	10.33 ± 0.20	20.54 ± 0.15
Cup	Plastic	2.52 ± 0.05	6.88 ± 0.08

Values represent arithmetic means ± standard deviation (*n* = 5 replicates).

**Table 2:** The list of attributes used for the descriptive sensory analysis to evaluate four different tableware items.

Attribute	Definition	Characteristic
SPOON		
Visual perception:		
Evenness of color	The evenness of distribution of the color, not blotchy	Uneven/Blotchy—Even
Regularity of shape	Regularity of the shape of the spoon (regular, not distorted, not bent, not curved)	Distorted/Bent—Regular
Feeling in the hands:		
Heaviness	The feeling in a part of the body of weighing a lot and being difficult to move	Light—Heavy
Flexibility	The ability of the spoon to bend without breaking when force is applied	Inflexible—Flexible
Slipperiness (handle/thumb)	Ease of sliding the thumb over the handle of the spoon	Drag—Slip
Edge sharpness (handle)	The amount of irregularity at the edges of the spoon handle or the possession of a pronounced, sharp edge that scratches or cuts	Smooth—Rough/Sharp
Feeling in contact with the lips:		
Edge sharpness (bowl)	The amount of irregularity at the edges of the spoon bowl or the possession of a pronounced, sharp edge that scratches or cuts	Smooth—Rough/Sharp
Surface roughness (bowl)	The overall roughness of the surface of the spoon bowl	Smooth—Rough
Feeling in contact with the tongue:		
Slipperiness (bowl/tongue)	Ease to slide tongue over the spoon bowl	Drag—Slip
Flavor intensity	The intensity of the overall flavor of the material	None—Intensive
FORK		
Visual perception:		
Evenness of color	The evenness of distribution of the color, not blotchy	Uneven/Blotchy—Even
Regularity of shape	Regularity of the shape of the fork (regular, not distorted, not bent, not curved)	Distorted/Bent—Regular
Feeling in the hands:		
Heaviness	The feeling in a part of the body of weighing a lot and being difficult to move	Light—Heavy
Flexibility	The ability of the fork to bend without breaking when force is applied	Inflexible—Flexible
Slipperiness (handle/thumb)	Ease to slide thumb over the handle of the fork	Drag—Slip
Edge sharpness (handle)	The amount of irregularity at the edges of the fork handle or the possession of a pronounced sharp edge that scratches or cuts	Smooth—Rough/Sharp
Feeling in contact with the lips:		
Edge sharpness (tines)	The amount of irregularity at the edges of the fork head (tines) or the possession of a pronounced sharp edge that scratches or cuts	Smooth—Rough/Sharp
Surface roughness (tines)	The overall roughness of the surface of the fork head	Smooth—Rough
Feeling in contact with the tongue:		
Slipperiness (tines/tongue)	Ease to slide tongue over the fork head	Drag—Slip
Flavor intensity	The intensity of the overall flavor of the material	None—Intensive
PLATE		
Visual perception:		
Evenness of color	The evenness of distribution of the color, not blotchy	Uneven/Blotchy—Even
Regularity of shape	Regularity of the shape of the plate (regular, not distorted, not bent, not curved)	Distorted/Bent—Regular
Bottom roughness	The amount of irregularity, protrusions, grains, or bumps that can be seen on the bottom of the plate	Smooth—Rough
Edge wrinkling		Flat—Wrinkled
Presence of deformities		None—Intensive
Feeling in the hands:		
Heaviness	The feeling in a part of the body of weighing a lot and being difficult to move	Light—Heavy
Flexibility	The ability of the plate to bend without breaking when force is applied	Inflexible—Flexible
Firmness	The force required to bend the plate	Soft—Firm
Edge sharpness	The amount of irregularity on the edge of the plate or possession of a pronounced sharp edge that scratches or cuts	Smooth—Rough/Sharp)
CUP		
Visual perception:		
Translucency	A phenomenon that occurs between the extremes of opaqueness and transparency, partial transparency. Possibility of transmitting and scattering the light at the same time.	Opaque—Transparent
Feeling in the hands:		
Heaviness	The feeling that a part of the body weighs a lot and is difficult to move.	Light—Heavy
Flexibility	The ability of the cup to bend without breaking when force is applied.	Inflexible—Flexible
Firmness	The force required to compress between fingers.	Soft—Firm
Crispy/Rustle sound	The noise/sound with which the cup crumples or fractures.	No sound—Crispy/Rustle
Feeling in contact with the lips:		
Roughness/Sharpness	The amount of irregularity on the rim of the cup or possession of a pronounced sharp rim that scratches or cuts the lips.	Smooth—Rough/Sharp
Graininess	Number of small grains/particles on surface below the rim.	Smooth—Grainy
Moistness	Sensation of the transfer of moisture from the lips to the surface of the glass.	Dry—Moist
Feeling in contact with the teeth:		
Sonority	The property of producing a ringing sound when struck with teeth.	Dull sound—Ringing sound

**Table 3:** Proportions of evoked emotional responses reported by participating students when tasting the four different meals served with tableware made of different materials.

Emotion Terms	Type of Tableware	Warm Animal-Based Meal—PORK Stew (*n* = 59)	Warm Plant-Based Meal—Peas (*n* = 61)	Cold Animal-Based Meal—Cheese-Meat (*n* = 62)	Cold Plant-Based Meal—Salads (*n* = 60)
Calm	Regular	0.695 (a)	0.705 (a)	0.887 (b)	0.817 (a)
	Wood	0.729 (a)	0.623 (a)	0.694 (a)	0.717 (a)
	Plastic	0.695 (a)	0.721 (a)	0.726 (a)	0.733 (a)
Nervous	Regular	-	-	0.065 (a)	0.033 (a)
	Wood	-	-	0.210 (b)	0.167 (b)
	Plastic	-	-	0.161 (ab)	0.100 (ab)
Good	Regular	0.847 (a)	0.770 (a)	-	0.867 (b)
	Wood	0.729 (a)	0.689 (a)	-	0.667 (a)
	Plastic	0.814 (a)	0.787 (a)	-	0.767 (ab)
Bad	Regular	-	-	-	0.017 (a)
	Wood	-	-	-	0.150 (b)
	Plastic	-	-	-	0.133 (ab)
Comfortable	Regular	0.831 (b)	0.705 (b)	0.839 (b)	0.850 (b)
	Wood	0.593 (a)	0.557 (a)	0.613 (a)	0.600 (a)
	Plastic	0.729 (ab)	0.721 (b)	0.694 (ab)	0.733 (ab)
Irritated	Regular	-	-	0.032 (a)	0.050 (a)
	Wood	-	-	0.194 (b)	0.150 (a)
	Plastic	-	-	0.145 (ab)	0.133 (a)
Energetic	Regular	0.644 (b)	0.639 (a)	0.742 (b)	0.700 (b)
	Wood	0.492 (a)	0.590 (a)	0.581 (ab)	0.517 (a)
	Plastic	0.576 (ab)	0.623 (a)	0.548 (a)	0.567 (ab)
Tormented	Regular	-	-	0.048 (a)	-
	Wood	-	-	0.210 (b)	-
	Plastic	-	-	0.161 (ab)	-
Interested	Regular	0.712 (a)	0.721 (b)	0.790 (b)	0.750 (b)
	Wood	0.593 (a)	0.508 (a)	0.629 (ab)	0.600 (ab)
	Plastic	0.729 (a)	0.689 (b)	0.597 (a)	0.550 (a)
Bored	Regular	-	-	-	0.050 (a)
	Wood	-	-	-	0.183 (b)
	Plastic	-	-	-	0.167 (ab)
Pleasant	Regular	0.831 (b)	0.787 (a)	0.855 (b)	0.833 (b)
	Wood	0.661 (a)	0.689 (a)	0.677 (a)	0.667 (a)
	Plastic	0.797 (ab)	0.754 (a)	0.758 (ab)	0.767 (ab)
Unpleasant	Regular	-	-	0.032 (a)	0.050 (a)
	Wood	-	-	0.177 (b)	0.167 (a)
	Plastic	-	-	0.129 (ab)	0.133 (a)
Satisfied	Regular	0.864 (b)	0.803 (a)	0.887 (b)	0.917 (b)
	Wood	0.712 (a)	0.672 (a)	0.677 (a)	0.617 (a)
	Plastic	0.831 (ab)	0.787 (a)	0.774 (ab)	0.767 (ab)
Unsatisfied	Regular	0.068 (a)	-	0.032 (a)	0.050 (a)
	Wood	0.169 (a)	-	0.210 (b)	0.200 (b)
	Plastic	0.068 (a)	-	0.161 (ab)	0.150 (ab)
Like it *	Regular	0.932 (b)	0.951 (b)	0.887 (b)	0.900 (b)
	Wood	0.508 (a)	0.574 (a)	0.387 (a)	0.417 (a)
	Plastic	0.559 (a)	0.639 (a)	0.565 (a)	0.583 (a)
Dislike it *	Regular	0 (a)	0.033 (a)	0.048 (a)	0.033 (a)
	Wood	0.322 (b)	0.262 (b)	0.419 (b)	0.383 (b)
	Plastic	0.220 (b)	0.180 (ab)	0.274 (b)	0.267 (b)
Blissful	Regular	0.627 (a)	0.689 (b)	0.694 (a)	0.683 (b)
	Wood	0.508 (a)	0.525 (a)	0.581 (a)	0.450 (a)
	Plastic	0.599 (a)	0.639 (ab)	0.613 (a)	0.633 (b)
Tame	Regular	0.508 (a)	0.525 (a)	0.677 (b)	0.550 (a)
	Wood	0.424 (a)	0.475 (a)	0.532 (a)	0.483 (a)
	Plastic	0.508 (a)	0.508 (a)	0.532 (a)	0.483 (a)
Eager/craving	Regular	0.593 (a)	0.738 (b)	0.710 (b)	0.583 (b)
	Wood	0.424 (a)	0.525 (a)	0.532 (a)	0.417 (a)
	Plastic	0.492 (a)	0.574 (a)	0.565 (ab)	0.433 (ab)
Merry/Joyful	Regular	0.763 (a)	0.721 (b)	0.790 (b)	0.717 (a)
	Wood	0.610 (a)	0.541 (a)	0.645 (ab)	0.567 (a)
	Plastic	0.644 (a)	0.639 (ab)	0.629 (a)	0.667 (a)
Enthusiastic	Regular	0.627 (b)	0.689 (a)	0.758 (b)	0.633 (b)
	Wood	0.458 (a)	0.557 (a)	0.516 (a)	0.433 (a)
	Plastic	0.542 (ab)	0.672 (a)	0.565 (a)	0.550 (ab)

The table shows the data only for the terms that were chosen in more than 10% of the total occurrences. Values in the same column within an emotion marked with the same letter in the brackets are not statistically different (*α* = 0.05). * the conversion of the 9-point hedonic data to binary data is explained in the Section 2.

**Table 4:** Hedonic ratings for meal samples served in different types of tableware sets.

Hedonic Attributes	Type of Tableware	Warm Animal-Based meal—Pork Stew (*n* = 59)	Warm Plant-Based Meal—Peas (*n* = 61)	Cold Animal-Based Meal—Cheese-Meat (*n* = 62)	Cold Plant-Based Meal—Salads (*n* = 60)
Taste of the meal	Regular	7.0 ± 1.6 (b)	7.0 ± 1.9	7.6 ± 1.8 (b)	7.1 ± 1.8 (b)
	Wood	6.3 ± 2.0 (a)	6.7 ± 2.1	6.8 ± 1.9 (a)	6.2 ± 2.2 (a)
	Plastic	6.6 ± 1.8 (a, b)	6.9 ± 2.9	7.1 ± 1.4 (a, b)	6.7 ± 1.9 (a, b)
Dishes used	Regular	8.1 ± 1.1 (b)	7.9 ± 1.5 (b)	8.1 ± 1.5 (b)	8.1 ± 1.7 (b)
	Wood	4.3 ± 2.4 (a)	4.6 ± 2.8 (a)	3.7 ± 2.6 (a)	3.9 ± 2.8 (a)
	Plastic	4.4 ± 2.5 (a)	5.0 ± 2.3 (a)	4.3 ± 2.2 (a)	4.7 ± 2.3 (a)
Cutlery used	Regular	8.2 ± 1.1 (b)	8.1 ± 1.5 (b)	8.1 ± 1.6 (b)	8.2 ± 1.8 (b)
	Wood	4.7 ± 2.7 (a)	4.5 ± 2.9 (a)	3.1 ± 2.5 (a)	3.6 ± 2.8 (a)
	Plastic	4.3 ± 2.3 (a)	5.0 ± 2.3 (a)	3.8 ± 2.2 (a)	4.2 ± 2.4 (a)
Served meal	Regular	7.5 ± 1.2 (b) *	7.7 ± 1.3 (b) *	7.8 ± 1.7 (b) ^NS^	7.7 ± 1.5 (b) *
	Wood	5.3 ± 2.2 (a) *	5.7 ± 2.3 (a) *	4.9 ± 2.2 (a) *	5.0 ± 2.2 (a) *
	Plastic	5.6 ± 1.9 (a) *	6.0 ± 1.8 (a) *	5.7 ± 1.9 (a) *	5.5 ± 2.1 (a) *

Values are the arithmetic mean ± standard deviation. Values in the same column within a hedonic attribute marked with the same letter in the brackets are not statistically different (*α* = 0.5). ‘NS’ = the mean hedonic score for ‘served meal’ is not statistically different (*α* = 0.05) from the corresponding hedonic score for ‘taste of the meal’ within the same column; ‘*’ = the mean hedonic score for ‘served meal’ is statistically different (*α* = 0.05) from the corresponding hedonic score for ‘taste of the meal’ within the same column.

**Table 5:** Effects of tableware type on intensity scores: results of one-way ANOVA and Tukey’s HSD multiple comparisons test (consumers were included as a random factor and the type of cutlery as a fixed factor).

Attributes	Type of Tableware	Warm Animal-Based Meal—Pork Stew (*n* = 59)	Warm Plant-Based Meal—Peas (*n* = 61)	Cold Animal-Based Meal—Cheese-Meat (*n* = 62)
Overall odor	Regular	4.9 ± 1.3 (b)	4.4 ± 1.3 (b)	4.5 ± 1.8 (a)
	Wood	4.1 ± 1.4 (a)	3.8 ± 1.7 (a)	4.3 ± 1.6 (a)
	Plastic	4.1 ± 1.3 (a)	3.9 ± 1.6 (a)	4.3 ± 1.8 (a)
Salty taste	Regular	4.4 ± 1.5 (b)	3.9 ± 1.4 (b)	4.5 ± 1.2 (a)
	Wood	3.7 ± 1.5 (a)	3.4 ± 1.7 (a)	4.4 ± 1.2 (a)
	Plastic	3.9 ± 1.5 (a)	3.4 ± 1.8 (a)	4.5 ± 1.4 (a)
Flavor of spices	Regular	4.9 ± 1.4 (b)	4.0 ± 1.4 (a)	4.0 ± 1.8 (a)
	Wood	4.3 ± 1.6 (a)	3.9 ± 1.5 (a)	3.8 ± 1.8 (a)
	Plastic	4.5 ± 1.4 (a, b)	4.0 ± 1.7 (a)	3.7 ± 1.9 (a)
Atypical flavor	Regular	1.2 ± 1.6 (a)	1.0 ± 1.8 (a)	1.0 ± 1.9 (a)
	Wood	3.1 ± 2.3 (b)	2.5 ± 2.3 (b)	2.3 ± 2.3 (b)
	Plastic	1.3 ± 1.6 (a)	1.2 ± 1.8 (a)	1.4 ± 1.8 (a)
Overall flavor	Regular	5.0 ± 1.3 (b)	5.3 ± 1.3 (b)	5.0 ± 1.2 (a)
	Wood	4.4 ± 1.6 (a)	4.7 ± 1.5 (a)	4.8 ± 1.4 (a)
	Plastic	4.4 ± 1.3 (a)	4.7 ± 1.6 (a)	4.6 ± 1.3 (a)

Values are the arithmetic mean ± standard deviation. Values in the same column within a sensory attribute marked with the same letter in the brackets are not statistically different (*α* = 0.5). Note: The salad portions were not evaluated for the intensity of selected sensory attributes.

## Data Availability

The data presented in this study is available upon request from the corresponding author.

## Supplementary Materials

The following supporting information can be downloaded at https://www.mdpi.com/article/10.3390/foods14183151/s1, Table S1: List of emotions and moods that were used for the current study, Table S2: The results of the survey on the suitability of disposable tableware made of different materials at large events. The table shows the rank sums (n = 242 consumers; rank 1 was assigned to the most acceptable type of material and rank 4 to the least acceptable) together with the results of the Friedman analysis and the LSD multiple comparison procedure for rank sums; Table S3: The results of Tukey’s HSD multiple pairwise comparison test (α = 0.5) was performed after ANOVA on the standardized descriptive data of SPOONS made from three different materials. Attribute intensities increase progressively from subset 1 to subset 3, Table S4: The results of Tukey’s HSD multiple pairwise comparison test (α = 0.5) was performed after ANOVA on the standardized descriptive data of FORKS made from three different materials. Attribute intensities increase progressively from subset 1 to subset 3; Table S5: The results of Tukey’s HSD multiple pairwise comparison test (α = 0.5) was performed after ANOVA on the standardized descriptive data of PLATES made from three different materials. Attribute intensities increase progressively from subset 1 to subset 3; Table S6: The results of Tukey’s HSD multiple pairwise comparison test (α = 0.5) was performed after ANOVA on the standardized descriptive data of CUPS made from three different materials. Attribute intensities increase progressively from subset 1 to subset 3.
