# Supplementary material for: The Sensory and Emotional Response to Different Tableware Materials
Source: Foods. 2025 Sep 9;14(18):3151. doi: 10.3390/foods14183151 (PMC12469804; doi:10.3390/foods14183151)
Supplement: Supplementary file 1 [file foods-14-03151-s001.zip › Supplementary Materials.pdf]

Supplementary Table S1. List of emotions and moods that were used for the current study.

Moods and emotions			
	Negative		Positive
1	Unsatisfied	16	Satisfied
2	Bad	17	Good
3	Sad	18	Mery/Joyful
4	Bored	19	Interested
5	Unpleasant	20	Pleasant
6	Irritated	21	Comfortable
7	Tormented	22	Energetic
8	Pleased	23	Eager/Craving
9	Aggressive	24	Tame
10	Angry	25	Friendly
11	Worried	26	Relaxed
12	Nervous	27	Calm
13	Miserable	28	Blissful
14	Disgusted	29	Enthusiastic
15	Dislike it	30	Like it

Supplementary Table S2. The results of the survey on the suitability of disposable tableware made of different materials at large events. The table shows the rank sums (n = 242 consumers; rank 1 was assigned to the most acceptable type of material and rank 4 to the least acceptable) together with the results of the Friedman analysis and the LSD multiple comparison procedure for rank sums.

	Homogenous subsets at $\alpha = 0.01$.		
Type of material	I (the most acceptable)	II	III (the least acceptable)
Hard plastic	378		
Soft plastic		604	
Wood		640	
Paper			798

Supplementary Table S3**.** The results of Tukey's HSD multiple pairwise comparison test (α=0.5) was performed after ANOVA on the standardized descriptive data of SPOONS made from three different materials. Attribute intensities increase progressively from subset 1 to subset 3.

Evenness of color

Subset 1	Subset 2
Wood	
	Plastic
	Steel

Regularity of shape

Subset 1	Subset 2	Subset 3
Wood		
	Plastic	
		Steel

Heaviness

Subset 1	Subset 2
Plastic	
Wood	
	Steel

Flexibility

Subset 1	Subset 2	Subset 3
Steel		
	Wood	
		Plastic

Slipperiness (handle/thumb)

Subset 1
Wood
Plastic
Steel

Edge sharpness (handle)

Subset 1	Subset 2
Steel	
Wood	Wood
	Plastic

Edge sharpness (bowl)

Subset 1	Subset 2
Steel	
Wood	Wood
	Plastic

Surface roughness (bowl)

Subset 1	Subset 2
Steel	
Plastic	
	Wood

Slipperiness (bowl/tong)

Subset 1	Subset 2
Wood	
	Plastic
	Steel

Flavor

Subset 1	Subset 2
Plastic	
Steel	
	Wood

Supplementary Table S4**.** The results of Tukey's HSD multiple pairwise comparison test (α=0.5) was performed after ANOVA on the standardized descriptive data of FORKS made from three different materials. Attribute intensities increase progressively from subset 1 to subset 3.

Evenness of color

Subset 1	Subset 2	
Wood		
	Plastic	
		Steel

Regularity of shape

Subset 1	Subset 2
Wood	
Plastic	Plastic
	Steel

Heaviness

Subset 1	Subset 2
Plastic	
Wood	
	Steel

Flexibility

Subset 1	Subset 2
Steel	
	Wood
	Plastic

Slipperiness (handle/thumb)

Subset 1
Wood
Plastic
Steel

Edge sharpness (handle)

Subset 1	Subset 2
Steel	
Wood	Wood
	Plastic

Edge sharpness (tines)

Subset 1	Subset 2
Wood	
Steel	Steel
	Plastic

Surface roughness (tines)

Subset 1	Subset 2
Steel	
Plastic	
	Wood

Slipperiness (tines/tong)

Subset 1	Subset 2
Wood	
	Plastic
	Steel

Flavor intensity

Subset 1	Subset 2
Steel	
Plastic	
	Wood

Supplementary Table S5**.** The results of Tukey's HSD multiple pairwise comparison test (α=0.5) was performed after ANOVA on the standardized descriptive data of PLATES made from three different materials. Attribute intensities increase progressively from subset 1 to subset 3.

Evenness of color

Subset 1	Subset 2
Plastic	
Cardboard	
	Ceramic

Regularity of shape

Subset 1	Subset 2
Cardboard	
Plastic	
	Ceramic

Bottom roughness

Subset 1	Subset 2
Ceramic	
Cardboard	
	Plastic

Edge wrinkling

Subset 1	Subset 2	Subset 3
Ceramic		
	Plastic	
		Cardboard

Presence of deformities

Subset 1	Subset 2
Ceramic	
	Cardboard
	Plastic

Heaviness

Subset 1	Subset 2	Subset 3
Plastic		
	Cardboard	
		Ceramic

Flexibility

Subset 1	Subset 2
Ceramic	
	Cardboard
	Plastic

Firmness

Subset 1	Subset 2
Cardboard	
Plastic	
	Ceramic

Edge sharpness

Subset 1	Subset 2
Ceramic	
	Cardboard
	Plastic

Supplementary Table S6**.** The results of Tukey's HSD multiple pairwise comparison test (α=0.5) was performed after ANOVA on the standardized descriptive data of CUPS made from three different materials. Attribute intensities increase progressively from subset 1 to subset 3.

Translucency

Subset 1	Subset 2	Subset 3
Cardboard		
	Plastic	
		Glass

Heaviness

Subset 1	Subset 2	Subset 3
Plastic		
	Cardboard	
		Glass

Flexibility

Subset 1	Subset 2
Glass	
	Plastic
	Cardboard

Firmness

Subset 1	Subset 2	Subset 3
Plastic		
	Cardboard	
		Glass

Crispy/Rustle sound

Subset 1	Subset 2	Subset 3
Glass		
	Cardboard	
		Plastic

Roughness/Sharpness

Subset 1	Subset 2	Subset 3
Glass		
	Cardboard	
		Plastic

Graininess

Subset 1	Subset 2
Glass	
	Plastic
	Cardboard

Moistness

Subset 1
Plastic
Cardboard
Glass

Sonority

Subset 1	Subset 2
Cardboard	
	Plastic
	Glass
